# Tumor Suppressor miR-584-5p Inhibits Migration and Invasion in Smoking Related Non-Small Cell Lung Cancer Cells by Targeting YKT6

**DOI:** 10.3390/cancers13051159

**Published:** 2021-03-08

**Authors:** Saet Byeol Lee, Young Soo Park, Jae Sook Sung, Jong Won Lee, Boyeon Kim, Yeul Hong Kim

**Affiliations:** 1 Cancer Research Institute, Korea University College of Medicine, Goryeodae-ro 73, Seongbuk-gu, Seoul 02841, Korea; akdltmxkf@korea.ac.kr (S.B.L.); difco@korea.ac.kr (Y.S.P.); potato23@korea.ac.kr (J.W.L.); alice_1989@korea.ac.kr (B.K.); 2 BK21 Plus Program, Korea University College of Medicine, Goryeodae-ro 73, Seongbuk-gu, Seoul 02841, Korea; 3 K-MASTER Cancer Precision Medicine Diagnosis and Treatment Enterprise, Korea University Anam Hospital, Goryeodae-ro 73, Seongbuk-gu, Seoul 02841, Korea; jsssung@korea.ac.kr; 4 Department of Biomedical Sciences, Korea University College of Medicine, Goryeodae-ro 73, Seongbuk-gu, Seoul 02841, Korea; 5 Department of Oncology/Hematology, Korea University Anam Hospital, Korea University College of Medicine, Goryeodae-ro 73, Seongbuk-gu, Seoul 02841, Korea

**Keywords:** smoking, non-small cell lung cancer, methylation, miR-584-5p, YKT6

## Abstract

**Simple Summary:**

Cigarette smoke is a major carcinogen that causes lung cancer and induces DNA methylation. DNA methylation regulates the expression of microRNA (miRNAs), which are important regulators of cancer biology. However, the association between smoking and miRNAs has not been fully elucidated in smoking-related lung carcinogenesis. In this study, we found that miR-584-5p expression was downregulated with cancer progression using a lung carcinogenesis model cell line. Moreover, we demonstrated that miR-584-5p is downregulated by the methylation of its promoter region and that it suppresses migration and invasion by targeting YKT6 in smoking-related non-small cell lung cancer (NSCLC) cells. Our results provide a better understanding of the underlying changes in miRNA expression in smoking-related lung carcinogenesis and suggest that miR-584-5p is a potential molecular biomarker for smoking-related NSCLC.

**Abstract:**

Cigarette smoke (CS) affects the expression of microRNAs (miRNAs), which are important regulators of gene expression by inducing DNA methylation. However, the effects of smoking on miRNA expression have not been fully elucidated in smoking-related lung carcinogenesis. Therefore, in this study, to investigate the change of miRNA expression pattern and to identify tumor suppressor miRNAs by smoking in lung carcinogenesis, we used lung carcinogenesis model cell lines that, derived from a murine xenograft model with human bronchial epithelial cells (BEAS-2B), exposed CS or not. The microarray analysis revealed that miR-584-5p expression was downregulated with cancer progression in lung carcinogenesis model cell lines. We confirmed by pyrosequencing that the methylation level of the miR-584-5p promoter increased with cancer progression. In vitro and in vivo experiments showed that miR-584-5p suppressed migration and invasion in non-small cell lung cancer (NSCLC) cells by targeting YKT6. Furthermore, we showed that high level of YKT6 was associated with a poor survival rate in NSCLC patients with a history of smoking. These results suggest that miR-584-5p acts as a tumor suppressor and is a potential molecular biomarker for smoking-related NSCLC.

## 1. Introduction

Lung cancer (LC) is one of the most common cancers worldwide [1,2] and is classified into two main categories: small cell lung cancer (SCLC) and non-small cell lung cancer (NSCLC). NSCLC accounts for about 85% of all lung cancers and is classified into three main histological subtypes: adenocarcinoma, squamous cell carcinoma, and large cell carcinoma [3,4]. LC is caused by factors such as smoking, exposure to radon, exposure to asbestos, and air pollution. Among these, smoking is the major risk factor for development of LC. In addition, the lungs are the major organ affected by cigarette smoke, and smoking accounts for 87% of deaths from LC [5]. Cigarette smoke (CS) contains a complex mixture of about 5000 chemicals, including nicotine, tar, benzo(a)pyrene (BaP), acetaldehyde, and nitric oxide (NO). Of these, more than 60 CS compounds are well known carcinogens [6]. Despite the proven relationship between smoking and increased risk of LC, the underlying mechanisms of how smoking contributes to lung carcinogenesis are not completely understood [7].

Smoking is known to contribute to carcinogenesis by causing epigenetic changes, such as DNA methylation and histone modification [8]. The DNA methylation of promotor regions regulates gene expression by suppressing the transcription of protein-coding genes and microRNA-coding genes [9]. Although DNA methylation is essential for the normal functioning of cells, abnormal hypermethylation and hypomethylation can contribute to cancer [10]. For example, DNA methylation of the promoter regions of tumor suppressor genes can contribute to tumor formation [11,12]. Therefore, an assessment of the methylation status of the promoter regions of specific genes has been proposed as a method for the early detection of cancer [13].

MicroRNAs (miRNAs) are small non-coding RNAs that regulate target gene expression by binding to complementary bases in the 3′ untranslated region (UTR) of their target mRNAs [14,15]. miRNAs regulate various biological processes, including those involved in critical pathways related to cell proliferation, apoptosis, metastasis, and invasion [16]. Several studies have shown that the expression level of specific miRNAs varies according to disease stage [17]. However, no prior studies have examined miRNA expression pattern in lung carcinogenesis due to smoking. Therefore, we analyzed changes in the miRNA expression pattern and degree of methylation using a lung carcinogenesis model cell line (Table 1). As a result, we identified a tumor suppressor miRNA that plays an important role in smoking-related lung cancer and investigated its biological role in smoking-related NSCLC cells.

## 2. Materials and Methods

### 2.1. Cell Culture and Transfection

Human lung cancer cell lines (H1703, A549, H522, H1299, and H358) were acquired from American Type Culture Collection (ATCC) (Manassas, VA, USA) and cultured in Roswell Park Memorial Institute (RPMI)—1640 with 1% of antibiotics and 10% of fetal bovine serum (FBS) (HyClone, Logan, UT, USA). To establish stable cell lines overexpressing miR-584-5p, A549 cells were infected with Lv12-u6/miR-584-5p or the negative control (Genepharma, Shanghai, China). Forty-eight hours after infection, cells were treated with 1-μg/mL puromycin to select transformed cells. BEAS-2B (human bronchial epithelial cells) and lung carcinogenesis model cell lines (1799 cells, normal immortalized cells; 1198 cells, transformed cells; and 1170I, tumorigenic cells) were gifts from Dr. Curtis Harris (National Institutes of Health, Bethesda, MD, USA) [20] and Dr. Andres Klein-Szanto (Fox Chase Cancer Center, Philadelphia, PA, USA) [19]. Culture conditions for BEAS-2B and lung carcinogenesis model cell lines were described in a previous study [21]. All cells were incubated at 5% CO_2_ and 37 °C. miR-584-5p mimics and negative controls were generated by Ambion (Thermo Fisher Scientific, Austin, TX, USA). Negative control small interfering RNA (siRNA) and YKT6 siRNA were purchased from Santa Cruz Biotechnology (Santa Cruz, CA, USA). miRNA and siRNA were used at 20 nM and transfected into cells with Lipofectamine RNAiMAX (Invitrogen, Carlsbad, CA, USA), according to the manufacturer’s instructions. pCMV6 control plasmid and pCMV6-YKT6 were purchased from Origene (Rockville, MD, USA). In rescue experiments, cells were co-transfected with miR-NC or miR-584-5p mimics and pCMV6 control vector or YKT6 overexpression vector using Lipofectamine 3000 (Invitrogen, Carlsbad, CA, USA).

### 2.2. Treatment with Demethylation Agent

Cells were treated with 5-aza-2′-deoxycytidine (5-Aza-dC) or DMSO (Sigma-Aldrich, St. Louis, MO, USA) as the control for 72 h. Then, cells were seeded for migration and invasion assays. Cell pellets were stored at −80 °C for DNA and RNA experiments.

### 2.3. Microarray Analysis

MicroRNA expression in lung carcinogenesis model cell lines (1799, 1198, and 1170I) was analyzed using Affymetrix’s Gene Chip miRNA Array 4.0 (Affymetrix, Santa Clara, CA, USA). Total RNA (500 ng), including miRNA, was biotin HSR-labeled using FlashTag. Samples were hybridized to the Affymetrix miRNA microarray (DNA link, Seoul, Korea) in a hybridization oven according to the protocols provided by the manufacturer.

### 2.4. Pyrosequencing Analysis

Genomic DNA was extracted from cell pellets using the QIAamp DNA Blood Mini kit (QIAGEN, Hilden, Germany) and quantified by Nanodrop (NanoDrop Technologies, Wilmington, DE, USA). Genomic DNA (300 ng) was used in bisulfite conversion reactions with the Lightning kit (Zymo Research, Irvine, CA, USA), according to the manufacturer’s protocols. Pyrosequencing was performed according to the manufacturer’s protocols (PyroGold Reagent kit, QIAGEN) by a service provider (Genomictree, Daejeon, Korea). PCR conditions consisted of incubation at 95 °C for 10 min, followed by 45 cycles of 95 °C for 30 s, 55 °C for 30 s, and 72 °C for 30 s and then a final annealing and extension step at 72 °C for 5 min. Primer sequences were as follows: primer 1, miR-584-5p (-730)-F: ATTAAAGGTTGTATTGTGTATTGA, miR-584-5p (-730)-R: biotin- CACCCATATATATACCATCCTAC, and miR-584-5p (-730)-S: TTGTGTATTGAGTAGGTT and primer2, miR-584-5p (-730)-F: ATTAAAGGTTGTATTGTG TATTGA, miR-584-5p (-730)-R: biotin-CACCCATATATATACCATCCTAC, miR-584-5p (-730)-S: TTGTGTATTGAGTAGGTT, primer 3, miR-584-5p (-583)-F: GGTTAGGGTA GGATGGTATATATATGG, miR-584-5p (-583)-R: biotin-CCCAACAAATCCCTAAAC CTCTA, and miR-584-5p (-583)-S: GGTGGTTGTTTTTGTAT.

### 2.5. Gene Expression Omnibus (GEO) Database Analysis

Microarray data (https://www.ncbi.nlm.nih.gov/geo/; accession numbers GSE74190 and GSE19945) were used in this study to evaluate the expression levels of miR-584-5p in various tumor types. The GSE74190 dataset includes data from 18 small cell lung carcinoma (SCLC) tissue samples, 29 squamous cell carcinoma (SQ) tissue samples, and 44 adjacent normal tissue samples, while the GSE19945 dataset contains microarray data from 35 SCLC tissue samples, five SQ tissue samples, and eight adjacent normal tissue samples. We also used microarray data (accession number GSE31210) to evaluate the association between the expression level of YKT6 mRNA and survival rate. The GSE31210 dataset contains data from lung tumor and normal tumor tissue samples. An independent Student’s *t*-test was performed to determine the significant difference in the miR-584-5p expression between lung cancer tissues and adjacent normal control tissues. *p* < 0.05 was considered statistically significant.

### 2.6. RNA Isolation and Real-Time RT-PCR

Total RNA was isolated using Qiazol reagent (QIAGEN). miRNA was purified and extracted using the miRNeasy Mini kit (QIAGEN) according to the manufacturer’s recommendations. Complementary DNA (cDNA) synthesis was performed using the TaqMan™ MicroRNA reverse transcription kit (Applied Biosystems, Foster City, CA, USA), and TaqMan real time-PCR was carried out according to the manufacturer’s instructions (Applied Biosystems). The expression of YKT6 mRNA was measured by SYBR Green quantitative PCR (Applied Biosystems). The expression of miR-584-5p was normalized to that of RNU6B, and the mRNA expression of YKT6 was normalized to that of β-actin.

### 2.7. Dual-Luciferase Reporter Assay

For the dual-luciferase reporter assay, the cells were seeded in 96-well plates at a number that reached confluency after a 72-h incubation. Then, the cells were co-transfected with 20-nM miRNA mimics or negative control miRNAs and 500 ng of pGL3-wt-YKT6 3′UTR or pGL3-mut-YKT6 3′UTR using Lipofectamine 3000. Forty-eight hours after transfection, luciferase activities were measured using the Dual-Glo Luciferase Assay System (Promega, Madison, WI, USA).

### 2.8. Wound-Healing Assay

For the dual-luciferase reporter assay, cells were seeded in 96-well plates at a number that ensured confluency after a 72-h incubation. Then, cells were co-transfected with 20-nM miRNA mimics or negative control miRNAs and 500 ng of pGL3-wt-YKT6 3′UTR or pGL3-mut-YKT6 3′UTR using Lipofectamine 3000. Forty-eight hours after transfection, luciferase activities were measured using the Dual-Glo Luciferase Assay System (Promega).

### 2.9. Trans-Well Assays

Invasion assays were carried out in 24-well Transwell chambers (Corning Costar Corp, Corning, NY, USA). Forty-eight hours after transfection, 1 × 10^5^ cells were seeded in the upper chamber in 200 μL serum-free medium, whereas the bottom chamber was filled with 750 μL 10% FBS medium. Twenty-four and 48 h later, respectively, the two chambers were washed and wiped off, and cells were fixed with 4% paraformaldehyde and 100% methanol. Next, chambers were stained with 0.1% crystal violet. Cells were counted and photographed in five randomly selected fields.

### 2.10. Western Blot Analysis

Cells were harvested and lysed in RIPA buffer with a protease inhibitor cocktail (1183170001, Roche, Hvidovre, Denmark) and phosphatase inhibitor cocktail (04906837001, Roche, New York, NY, USA). Total protein lysates were separated by 10% SDS-PAGE and transferred to Nitrocellulose membranes (66485, Pall Corporation, Port Washington, NY, USA). Membranes were then incubated with primary antibodies at 4 °C overnight. Subsequently, membranes were incubated with secondary antibodies at room temperature. Western blot analyses were carried out using the following antibodies: YKT6 (cat# sc-365732, Santa Cruz Biotechnology), matrix metalloproteinase 9 (MMP-9) (cat# 13667, Cell Signaling Technology, Danvers, MA, USA), and β-actin (cat# A5316, Sigma-Aldrich, St. Louis, MO, USA). Signals were visualized by enhanced chemiluminescence assays (Bio-Rad, Hercules, CA, USA).

### 2.11. Animal Studies

BALB/c athymic nude mice (3 to 4 weeks old) were purchased from Orient Bio Animal Center (Seongnam, Korea). All animal experiments were performed in accordance with the International Animal Care Use Committee (IACUC) of Korea University College of Medicine (IACUC approval No. KOREA-2019-0122-C1, date 8 January 2020).

#### 2.11.1. In Vivo Tumorigenicity Assays

Ten mice were randomly divided into two groups (*n* = 5): an Lv-miR-NC group and an Lv-miR-584-5p-overexpression group. A total of 1 × 10^6^ A549 cells transfected with Lv-miR-584-5p (or Lv-miR-NC) in 200 μL of PBS and Matrigel (356231, Corning Costar Corp) (1:1) mixture was injected subcutaneously into the flanks of mice to generate xenograft tumors. Tumor growth and weights were monitored every day. After thirty-two days, mice were sacrificed, and all tumor tissues were fixed with 4% paraformaldehyde. The expression of YKT6 in tumors was detected by qRT-PCR and immunohistochemistry assays.

#### 2.11.2. In Vivo Metastasis Assays

A549 cells were transfected with Lv-miR-NC or Lv-miR584-5p. Sixteen mice were randomly divided into two groups (*n* = 8). A total of 1 × 10^6^ transfected cells in PBS was injected through the tail vein. Seventy-nine days after injection, mice were sacrificed. Lung tissues were collected and fixed in 4% formalin.

### 2.12. Immunohistochemistry

Paraffin sections from tumor tissue samples in mice were prepared to detect the Ki-67(Dako, Glostrup, Denmark), YKT6 (cat# PA5-56565, Invitrogen). Immunostaining was performed using the Polink-2 Plus HRP Broad Kit with DAB detection system (GBI Labs, Mukilteo, WA, USA) according to the manufacturer’s protocols. The stained sections slides were visualized using the Slide Scanner (Axio Scan Z1, ZEISS, Oberkochen, Germany).

### 2.13. TUNEL Assay

We detected apoptosis in tumor tissue samples using an ApopTag Peroxidase in situ apoptosis detection kit (Merck Millipore, Darmstadt, Germany) according to the manufacturer’s instructions. The number of apoptosis-positive cells was counted under microscopy.

### 2.14. Kaplan–Meier Plot Analysis

Kaplan–Meier plot (https://kmplot.com/analysis/) analysis was utilized to determine the correlation between the mRNA expression of *YKT6* and survival outcomes in lung cancer patients with smoking experience. The Affymetrix ID corresponding to YKT6 is 217784_at.

### 2.15. Statistical Analysis

Each of our experiments was performed at least three times to ensure reproducibility. Data are expressed as mean ± standard deviation. A *p*-value < 0.05 was considered statistically significant. The significance of differences between groups was determined by Student’s *t*-test in GraphPad Prism 5.0 (GraphPad Inc., San Diego, CA, USA).

## 3. Results

### 3.1. miR-584-5p Expression Is Downregulated in Lung Carcinogenesis Model Cell Lines

**Figure 1:**
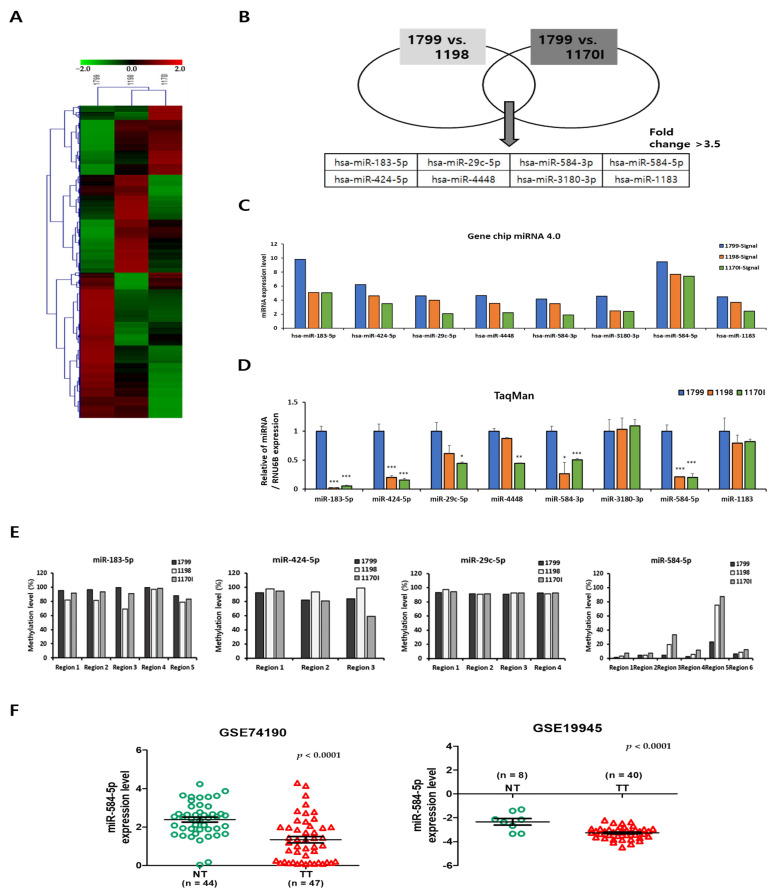
miR-584-5p was downregulated in lung carcinogenesis model cell lines. (**A**) Heat map clustering of differentially expressed microRNAs (miRNAs) in lung carcinogenesis model cell lines. Columns: lung carcinogenesis model cell lines (1799, 1198, and 1170I) and rows: miRNA (**B**) The Venn diagram shows the categories of the analysis group. Cigarette Smoke Condensate (CSC)-nonexposed, immortalized 1799 cells were compared with CSC-exposed, transformed 1198 cells, and CSC-nonexposed, immortalized 1799 cells were compared with CSC-exposed, tumorigenic 1170I cells. The eight miRNAs shown below the diagram are common miRNAs that showed decreased expression in 1198 cells compared to 1799 cells and in 1170I cells compared to 1799 cells. Threshold of >3.5-fold change and *p* < 0.05 were used to determine the significantly regulated miRNAs. (**C**) Microarray analysis of the expression of the eight miRNAs in lung carcinogenesis model cell lines. (**D**) qRT-PCR analysis of the eight miRNAs in lung carcinogenesis model cell lines; * *p* < 0.05, ** *p* < 0.01, and *** *p* < 0.001. (**E**) Methylation levels of the eight miRNAs in lung carcinogenesis model cell lines as determined by pyrosequencing. (**F**) Microarray analysis of miR-584-5p expression in tissues of lung cancer patients (https://www.ncbi.nlm.nih.gov/geo/; Gene Expression Omnibus (GEO) database accession numbers GSE74190 and GSE19945); *** *p* < 0.001.

We hypothesized that the expression of tumor suppressor miRNAs would decrease with progression of lung carcinogenesis caused by smoking. Thus, we analyzed the following cell lines, all of which were derived from a single cell line but represent different lung cancer development stages: (Cigarette Smoke Condensate or CSC-nonexposed, immortalized) 1799 cells vs. (CSC-exposed, transformed) 1198 cells and (CSC-nonexposed, immortalized) 1799 cells vs. (CSC-exposed, tumorigenic) 1170I cells. (Figure 1A,B). The microarray data analysis showed that the expression of eight miRNAs (miR-183-5p, miR-424-5p, miR-29c-5p, miR-4448, miR-584-3p, miR-3180-3p, miR-584-5p, and miR-1183) was significantly downregulated in these lung carcinogenesis model cell lines as the cancer progressed (Figure 1C). We then validated the expression of these eight miRNAs and confirmed four of the eight (miR-183-5p, miR-424-5p, miR -29c-5p, and miR-584-5p) were consistent with the analysis results of the microarray (Figure 1D). Furthermore, we evaluated the methylation levels of the promoter regions of these miRNAs in the lung carcinogenesis model cell lines. As shown in Figure 1E, among the four miRNAs, the methylation level of only the miR-584-5p promoter region increased significantly according to stage of cancer progression. These results indicate that the CpG regions methylation level of the promoter of miR-584-5p was negatively correlated with the expression of this miRNA in 1799, 1198, and 1170I cells. Additionally, we evaluated expression level of miR-584-5p in the Gene Expression Omnibus (GEO) database. We found that miR-584-5p expression was significantly downregulated in the lung tissue of patients with smoking-related lung cancer compared to adjacent normal tissues (GSE74190 and GSE19945) (Figure 1F).

### 3.2. miR-584-5p Regulates Migration and Invasion in Lung Carcinogenesis Model Cell Lines

**Figure 2:**
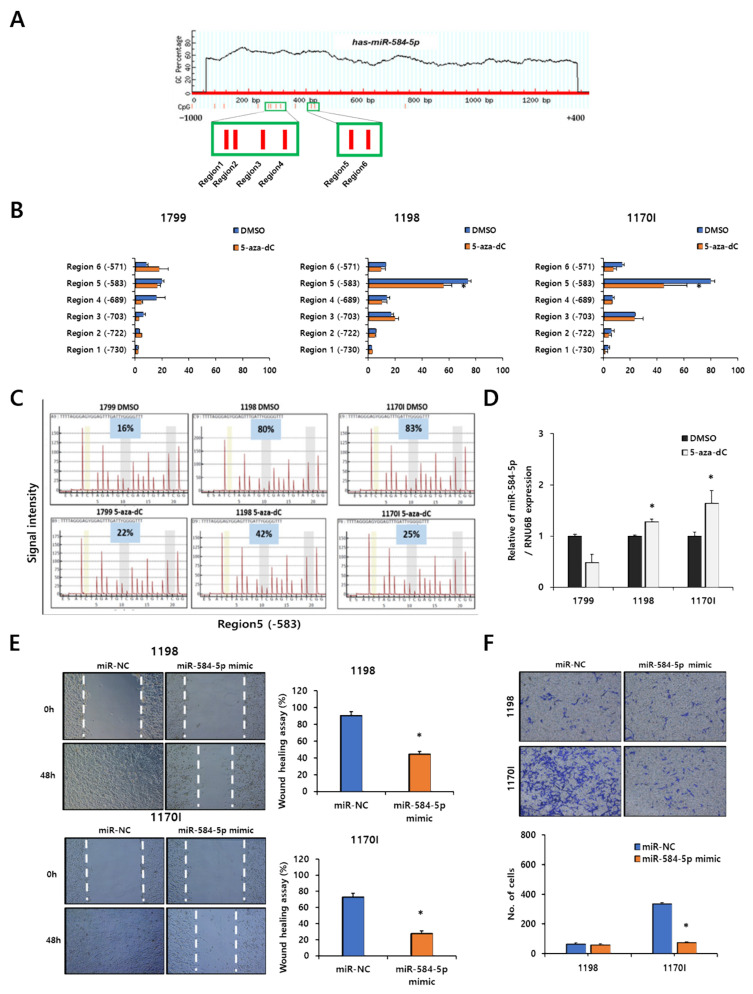
miR-584-5p downregulation is associated with hypermethylation in lung carcinogenesis model cell lines. (**A**) The CpG regions of miR-584-5p were predicted by MethPrimer (http://www.urogene.org), and the analyzed area is indicated with a green box. (**B**,**C**) The methylation levels and methylation signal intensity in lung carcinogenesis model cell lines with or without 5-aza-2′-deoxycytidine (5-aza-dC) treatment for 72 h by pyrosequencing. * *p* < 0.05. (**D**) The level of miR-584-5p expression in lung carcinogenesis model cell lines with or without 5-aza-2′-deoxycytidine (5-aza-dC) treatment for 72 h by qRT-PCR. * *p* < 0.05. Representative images and quantification of wound-healing (**E**) and Transwell (**F**) assays in 1198 and 1170I cells transfected with miR-584-5p mimic and the negative control. * *p* < 0.05.

We analyzed the methylation levels of six regions of the miR-584-5p promoter in lung carcinogenesis model cell lines. The 1170I cell line, which was the most highly methylated, showed the highest level of demethylation after treatment with the demethylation agent, 5-aza-2′-deoxycytidine (Figure 2A,B). In particular, among the CpGs, region 5 was the most demethylated after 5-aza-dC treatment (83% to 25%) in the 1170I cell line, followed by the 1198 cell line (80% to 42%), whereas the methylation levels of the 1799 cell line were not affected by the 5-aza-dC treatment (Figure 2C). These results indicate that downregulation of miR-584-5p was due to methylation of the promoter region of this miRNA and that region 5 is the major methylated CpG region in the promoter regions. In addition, the 5-aza-dC treatment restored miR-584-5p expression in 1198 and 1170I cell lines but not in the 1799 cell line (Figure 2D). Next, we investigated the migration and invasion ability of 1198 and 1170I cell lines in response to miR-584-5p overexpression. The overexpression of this miRNA in 1170I cells significantly reduced their migration and invasion ability, whereas miR-584-5p overexpression in 1198 cells decreased their migration ability only (Figure 2E,F).

### 3.3. Downregulation of miR-584-5p Expression Is Associated with Hypermethylation of Promoter CpG Island in Smoking-Related NSCLC Cells

**Figure 3:**
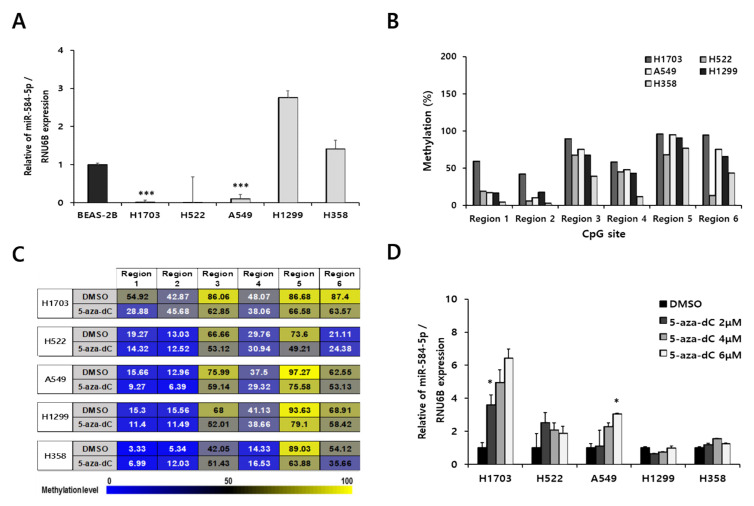
miR-584-5p expression was regulated by hypermethylation in smoking-related non-small cell lung cancer (NSCLC) cells. (**A**) qRT-PCR analysis of miR-584-5p expression level in smoking-related NSCLC cells; *** *p* < 0.001. (**B**) Basal methylation level of six CpGs of the miR-584-5p promoter in smoking-related NSCLC cells, as determined by pyrosequencing. (**C**) Methylation level of six CpGs of the miR-584-5p promoter in smoking-related NSCLC cells after 5-aza-2′-deoxycytidine (5-aza-dC) treatment, as determined by pyrosequencing. (**D**) qRT-PCR analysis of the miR-584-5p expression level in smoking-related NSCLC cells after 5-aza-2′-deoxycytidine (5-aza-dC) treatment; * *p* < 0.05.

We examined endogenous miR-584-5p levels in smoking-related NSCLC cell lines (H1703, H522, A549, H1299, and H358) and a normal human bronchial epithelial cell line (BEAS-2B). As shown in Figure 3A, miR-584-5p was significantly downregulated in the H1703, H522, and A549 cell lines but upregulated in the H1299 and H358 cell lines compared with the BEAS-2B cell line. Next, we investigated the methylation levels of those NSCLC cell lines. Most miR-584-5p promoter CpGs were more highly methylated in H1703 and A549 cells than in the other cell lines evaluated (Figure 3B). Consistent with our previous findings, region 5 of CpGs was the most strongly affected by 5-aza-dC treatment (Figure 3C). In addition, a significant dose-dependent increase of miR-584-5p expression was detected in H1703 and A549 cells (Figure 3D).

### 3.4. miR-584-5p Suppresses Migration and Invasion in Smoking-Related NSCLC Cell Lines

**Figure 4:**
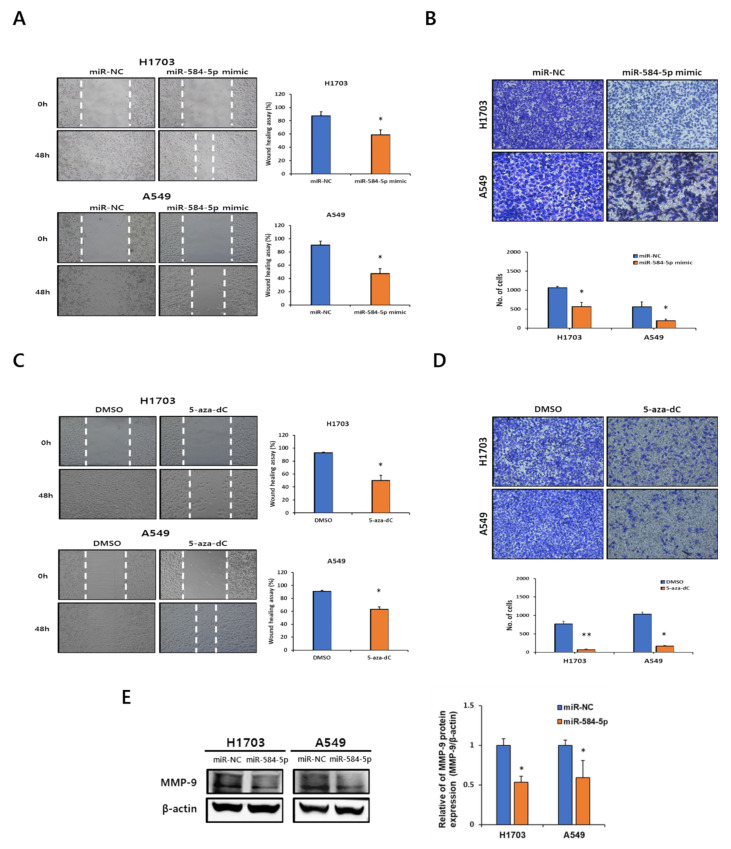
miR-584-5p inhibits the migration and invasion of smoking-related NSCLC cells in vitro. Representative images and quantification of the wound-healing assay (**A**) and Transwell assay (**B**) in H1703 and A549 cells transfected with miR-584-5p mimic or the negative control; * *p* < 0.05. Representative images and quantification of the wound-healing assay (**C**) and Transwell assay (**D**) in H1703 and A549 cells after the treatment with 5-aza-2′-deoxycytidine (5-aza-dC); * *p* < 0.05 and ** *p* < 0.01. (**E**) Effects of miR-584-5p mimic on the matrix metalloproteinase 9 (MMP-9) protein expression level as determined by the Western blot assay. Full Western Blot images can be found in Figure S1.

We next investigated the role of miR-584-5p in smoking-related-NSCLC cell migration and invasion in H1703 and A549 cells (two cell lines with a low expression of miR-584-5p and elevated methylation level). Consistent with our previous findings (Figure 2E,F), the migration and invasion abilities of H1703 and A549 cells were significantly decreased by miR-584-5p overexpression (Figure 4A,B). Additionally, we investigated changes in metastatic ability after 5-aza-dC treatment. Interestingly, 5-aza-dC treatment suppressed the migration and invasion of H1703 and A549 cells (Figure 4C,D). Several studies have reported that the inhibition of matrix metalloproteinases (MMPs) is involved in decreased cancer cell migration and invasion in NSCLC [22,23]. Thus, we evaluated the protein levels of the lung cancer metastasis-related factors, including MMP-9. As shown in Figure 4E, the expression of MMP-9 in smoking-related-NSCLC cells was decreased by an overexpression of miR-584-5p.

### 3.5. miR-584-5p Inhibits Tumor Growth and Lung Metastasis Abilities of NSCLC Cells In Vivo

**Figure 5:**
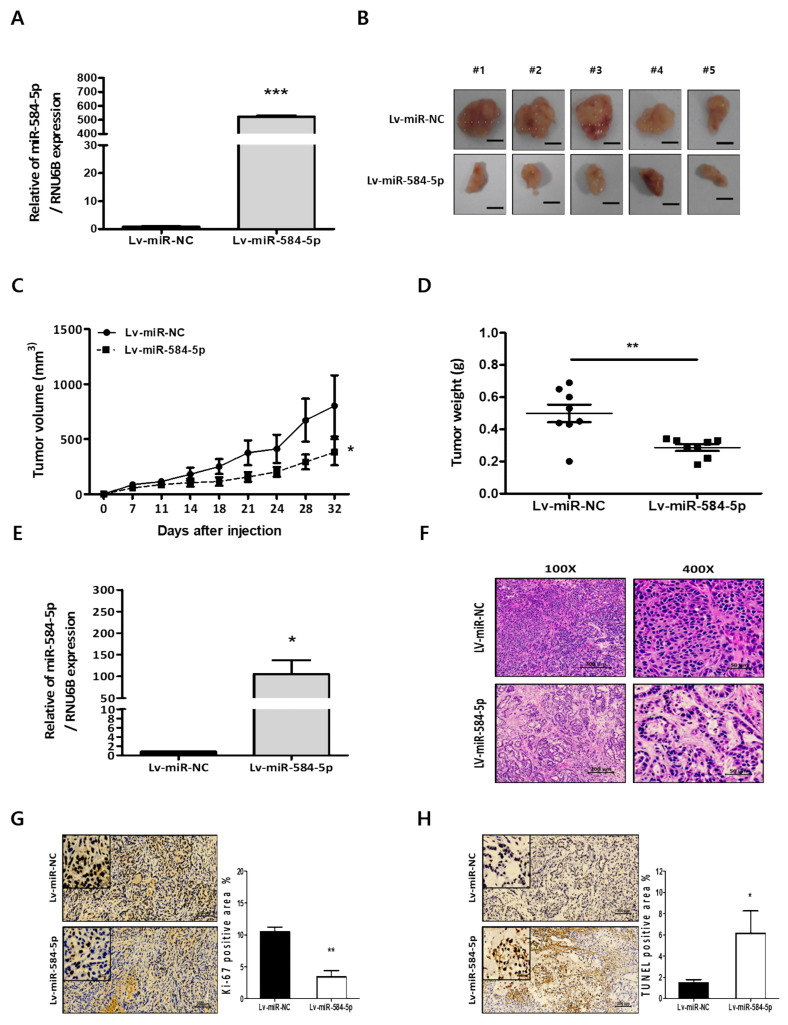
miR-584-5p suppresses tumor growth of smoking-related NSCLC cells in vivo. (**A**) Verification of the expression level of miR-584-5p by qRT-PCR in A549 cells infected with miR-584-5p-overexpressing lentivirus (Lv-miR-584-5p) or miR-negative control lentivirus (Lv-miR-NC); *** *p* < 0.001. (**B**) BALB/c nude mice (*n* = 5 mice per group) were subcutaneously injected with A549 cells infected with Lv-miR-584-5p or Lv-miR-NC. Representative images of tumors from BALB/c nude mice from the two groups. Scale bar represents 100 mm. (**C**,**D**) Tumor volumes and weights were measured in the two groups; * *p* < 0.05 and ** *p* < 0.01. (**E**) Verification of the expression level of miR-584-5p by qRT-PCR from xenograft tumors in the two groups; * *p* < 0.05. (**F**) Representative images of hematoxylin and eosin (H&E)-stained xenograft tumor sections from the two groups; magnification 100× and 400×, scale bar = 200 μm. Representative images are immunohistochemical (IHC) staining results for Ki-67 (**G**) and apoptotic cells (**H**) in xenograft tumor sections from the two groups; * *p* < 0.05 and ** *p* < 0.01. scale bar = 100 μm.

To examine the effects of miR-584-5p on tumor growth and lung metastasis abilities in vivo, we generated a stable cell line, which were infected with Lv-miR-584-5p in A549 cells and verified the expression of miR-584-5p (Figure 5A). Then, Lv-miR-NC-A549 and Lv-miR-584-5p-A549 cells were subcutaneously injected into nude mice. After 32 days, we identified that the average tumor volumes and weights were significantly reduced in the miR-584-5p-overexpressing group (Figure 5B–D). We also confirmed that the expression of miR-584-5p was more upregulated in the tumors of the miR-584-5p-overexpressing group than in the tumors of the miR-NC group (Figure 5E).

**Figure 6:**
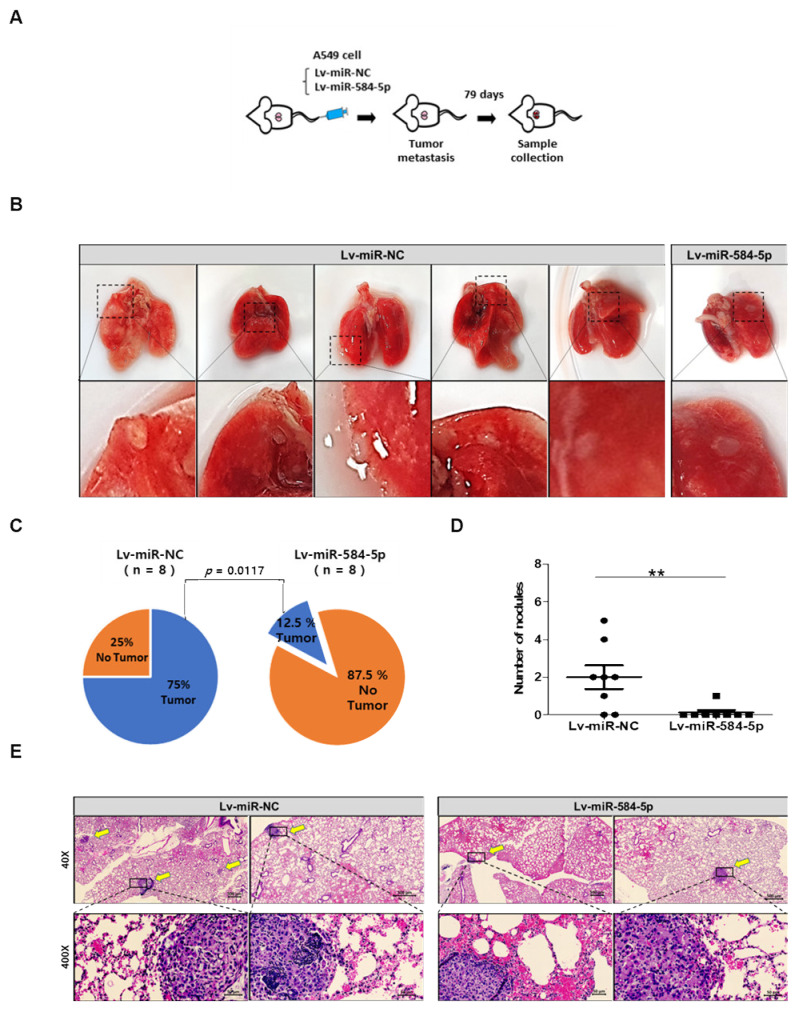
miR-584-5p inhibits the lung metastasis abilities of smoking-related NSCLC cells in vivo. (**A**) A549 cells transfected with miR-584-5p-overexpressing lentivirus or miR-control lentivirus were injected through the tail veil into BALB/c nude mice (*n* = 8 mice per group). Mice were sacrificed at 79 days after cell injection, and their lungs were collected. (**B**) Representative images of the lungs of the two groups of mice. (**C**) Pie graphs showing the incidence of metastatic nodules in lungs from the two groups of mice. (**D**) Numbers of metastatic lung nodules in the two groups; ** *p* < 0.01. (**E**) Representative images of H&E-stained tumor sections from lungs of mice in the two groups. Magnification, 40× and 400×. Arrows indicate metastatic lung nodules.

Hematoxylin and eosin (H&E) staining of tumor samples showed that miR-584-5p expression impaired tumor formation in the miR-584-5p-overexpressing group compared with that of the miR-NC group (Figure 5F). Ki-67 expression as a proliferation marker was decreased in miR-584-5p-overexpressing group tumors. Conversely, TUNEL staining as a marker of apoptosis was increased in miR-584-5p-overexpressing tumors compared to miR-NC tumors (Figure 5G,H). For the in vivo metastasis experiments, Lv-miR-NC-A549 and Lv-miR-584-5p-A549 cells were injected into mice through their tail veins (Figure 6A). Seventy-nine days after injection, these mice were sacrificed, and the metastatic foci in their lungs were assessed. We found that the overexpression of miR-584-5p significantly attenuated the incidence of lung metastasis and decreased the number of metastatic nodules (Figure 6B–D). H&E staining showed that lungs from miR-584-5p-overexpressing mice had fewer tumor nodules than lungs of miR-NC mice (Figure 6E).

### 3.6. YKT6 Is a Direct Target of miR-584-5p

**Figure 7:**
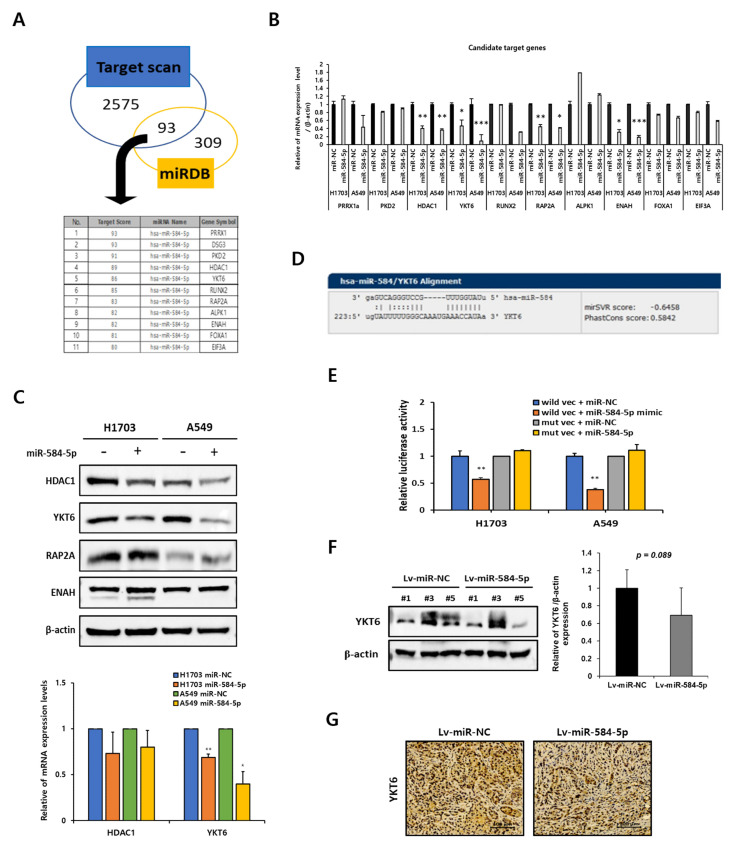
YKT6 is a direct target gene of miR-584-5p in smoking-related NSCLC cells. (**A**) miR DB (target score ≥ 80) and Targetscan database were used to analyze the target gene of miR-584-5p. Venn diagrams show groups of predicted target genes. (**B**) Verification of the mRNA expression of candidate target genes by qRT-PCR in H1703 and A549 cells transfected with miR-584-5p mimic or miR control; * *p* < 0.05, ** *p* < 0.01, and *** *p* < 0.001. (**C**) Verification of the protein expression of candidate target genes (HDAC1, YKT6, RAP2A, and ENAH) by Western blot assays in H1703 and A549 cells transfected with the miR-584-5p mimic or miR control; * *p* < 0.05 and ** *p* < 0.01. Full Western Blot images can be found in Figure S2. (**D**) Representative images of the has-miR-584/YKT6 binding sequence. (**E**) Relative luciferase activity was measured in H1703 and A549 cells transfected with the reporter vector containing wild-type or mutant-type YKT6 3′UTR (untranslated region), along with miR-584-5p mimic or miR control; ** *p* < 0.01. (**F**,**G**) Expression of YKT6 in xenograft tumor tissue was analyzed by Western blot and immunohistochemical (IHC) staining. Full Western Blot images can be found in Figure S3.

We searched two public bioinformatic databases (Target scan and miR DB) to predict the target genes of miR-584-5p and found 11 candidate genes with a target score of 80 or higher (Figure 7A). Among the candidate target genes, the endogenous mRNA expression of HDAC1, YKT6, RAP2A, and ENAH was reduced by more than 40% by overexpressed miR-584-5p in H1703 and A549 cells (Figure 7B). We also confirmed that the protein expression of YKT6 was significantly downregulated in H1703 and A549 cells by miR-584-5p (Figure 7C). Next, we searched for interaction sites between miR-584-5p and the 3′UTR of the YKT6 mRNA using miRbase (Figure 7D) and constructed a YKT6 3′UTR reporter luciferase assay system. Co-transfection experiments showed that miR-584-5p overexpression significantly decreased the luciferase activity of the YKT6 wild-type vector but not that of the mutant vector in H1703 and A549 cells (Figure 7E). Furthermore, we validated the results in the tumor samples from nude mice. As expected, the protein expression of YKT6 was slightly decreased in tumor tissues of the Lv-miR-584-5p group compared to the Lv-miR-NC group but did not reach statistical significance (Figure 7F). Consistent with these results, the IHC staining results showed that YKT6 expression was downregulated in tumor tissues of the Lv-miR-584-5p mice group (Figure 7G).

### 3.7. YKT6 Regulates Migration and Invasion in Smoking-Related NSCLC Cell Lines

**Figure 8:**
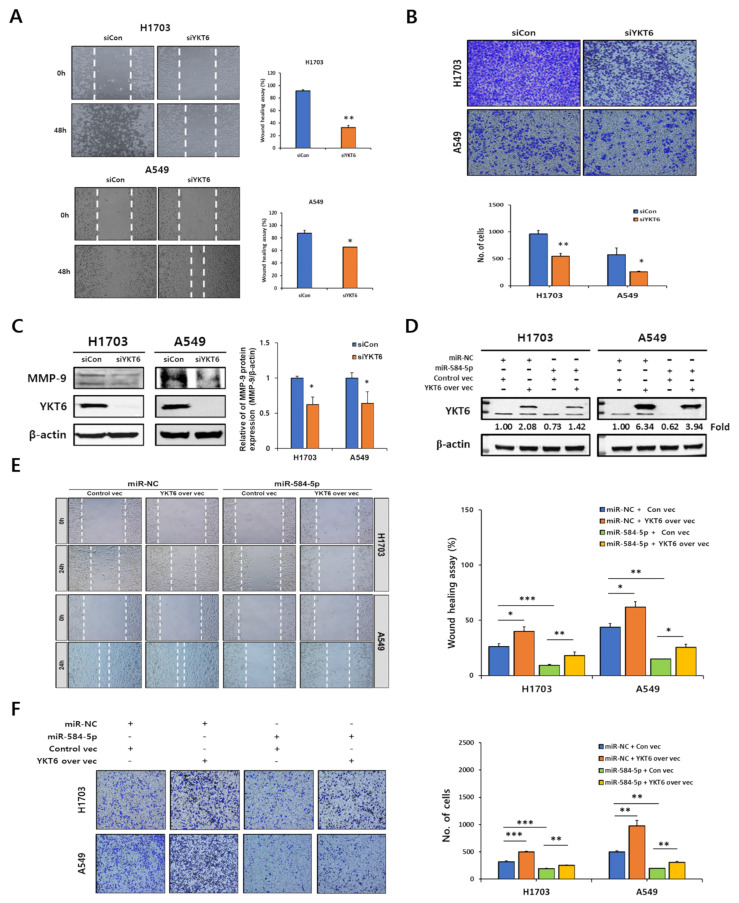
Depletion of YKT6 inhibited migration and invasion of smoking-related NSCLC cells. Representative images and quantification of the wound-healing assay (**A**) and Transwell assay (**B**) in H1703 and A549 cells transfected with siYKT6 or Control siRNA; * *p* < 0.05 and ** *p* < 0.01. (**C**) Verification of MMP-9 expression by Western blot assay in H1703 and A549 cells transfected with siYKT6 or Control siRNA. Full Western Blot images can be found in Figure S4 (**D**) Confirmation of transfection efficiency of H1703 and A549 cells by Western blot analysis. Full Western Blot images can be found in Figure S5. (**E**,**F**) Rescue of the migration and invasion abilities of H1703 and A549 cells by the exogenous expression of YKT6; * *p* < 0.05, ** *p* < 0.01, and *** *p* < 0.001.

We found that the depletion of YKT6 inhibited the migration and invasion abilities of smoking-related NSCLC cells (Figure 8A,B). Next, we assessed the protein level of MMP-9. Consistent with our earlier observations (Figure 4E), the protein level of MMP-9 was markedly decreased by the depletion of YKT6 in H1703 and A549 cells (Figure 8C). Rescue experiments revealed that YKT6 overexpression attenuated the inhibitory effect of miR-584-5p mimics on the YKT6 expression in H1703 and A549 cells (Figure 8D). We also investigated the effects of YKT6 overexpression on smoking-related NSCLC cell migration and invasion. The inhibitory effects of miR-584-5p overexpression on H1703 and A549 cell migration and invasion were attenuated by the expression of exogenous YKT6 (Figure 8E,F).

### 3.8. YKT6 Expression Level Is Associated with Survival Rate in Smoking-Related NSCLC Patients

**Figure 9:**
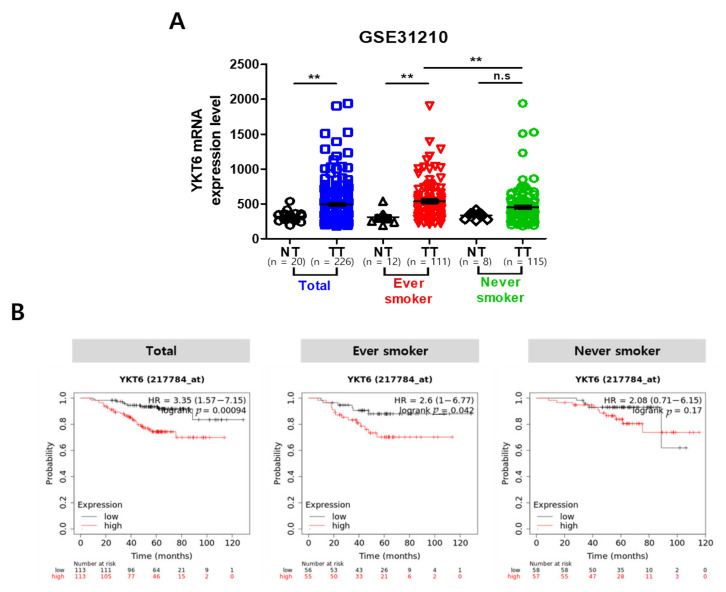
YKT6 expression level is associated with survival rate in NSCLC patients who smoked. (**A**) YKT6 expression level in NSCLC patient tumor tissue and adjacent normal tissue from the GSE31210 (GEO Database); ** *p* < 0.01. (**B**) Kaplan–Meier survival analysis shows that patients with a higher expression of YKT6 (217784_at) had a poorer overall survival than those with low YKT6 expression; ** *p* < 0.01, and n.s. = not significant.

We hypothesized that the expression of YKT6, the target gene of miR-584-5p, would be associated with smoking-related lung cancer. In the GSE31210 dataset, YKT6 mRNA expression was significantly upregulated in tumor tissues of ever-smokers compared to the tumor tissues of never-smokers in lung cancer patients (Figure 9A). Additionally, we analyzed the overall survival rate of lung cancer patients according to the YKT6 expression level using Kaplan–Meier plots. The group with elevated YKT6 expression had a low survival rate, and a significant *p*-value was obtained for smoking-related patients (Figure 9B).

## 4. Discussion

Many studies have demonstrated a correlation between smoking and miRNA expression in various cancers, including lung cancer [24,25,26]. In addition, previous reports have shown that cigarette smoking induces genetic changes through its effects on miRNAs [27]. Thus, miRNAs appear to play an important role in the development of smoking-related cancer. For example, cigarette smoking can induce miR-994 expression in oral cancer [28]. The expression of miR-21 was increased by cigarette smoke extract exposure in colorectal cancer [29], and the expression of this miRNA was elevated in esophageal cancers patients with consistent cigarette smoking [30]. miR-205 and miR-99a were shown to be downregulated in bladder cancer in smokers [31]. The expression of miR-486-5p is associated with the smoking-induced development of lung adenocarcinoma [25]. However, it is still insufficient to elucidate the specific miRNAs involved with the process of lung carcinogenesis caused by smoking. This is because most studies selected miRNAs that change with CSC exposure in normal or cancer cells or chose miRNAs based on smoking history [32,33]. In smoking-related carcinogenesis studies, cigarette smoke extract (CSE), cigarette smoke condensate (CSC), and whole cigarette smoke (WCS) are used to mimic the impacts of smoking [34]. CSE and CSC are mainly used in cell culture-based studies. CSE refers to the aqueous solution obtained by dissolving WCS in a cell culture medium or phosphate-buffered saline (PBS), while CSC refers to the solution obtained by collecting WCS in a filter pad and then dissolving it in organic solvents such as methanol or dimethyl sulfide side (DMSO) [35]. However, CSE or CSC does not reflect the composition of the gas released by smoking cigarettes, and these experimental methods do not reflect that CS affects the human lung by inhalation through the bronchus. In this study, we used three previously established lung carcinogenesis model cell lines—namely, 1799, 1198, and 1170I cells [19]. Although all these cell lines are derived from BEAS-2B cells, which are immortalized human normal bronchial epithelial cell lines, they represent different histological stages. For example, 1799 cells (immortalized) were derived from BEAS-2B cells exposed in vivo to beeswax only as a control, while 1198 (transformed) and 1170I (tumorigenic) cells were derived from BEAS-2B cells exposed in vivo to a beeswax pellet containing CSC. Importantly, the lung carcinogenesis model cell lines that we evaluated mimic the gradual changes that occur during human lung carcinogenesis induced by smoking [19]. Therefore, these cell lines are the best available in vitro model of lung carcinogenesis caused by smoking.

In the present study, we found that the expression of miR-584-5p is decreased during the process of lung carcinogenesis induced by cigarette smoking. Previous studies have reported that miR-584-5p expression is dysregulated in a variety of cancers, including hepatocellular carcinoma [36], medulloblastoma [37], gastric cancer [38], lung adenocarcinoma [39], and non-small cell lung cancer [40]. For example, Wei et al. [40] reported that miR-584-5p expression was downregulated in tissues of NSCLC patients and that the overexpression of miR-584-5p inhibited migration and invasion by targeting MMP-14. However, the mechanism by which miR-584-5p is downregulated in several cancers, including NSCLC cells, has not been identified. In this report, we showed that miR-584-5p expression is regulated by methylation in lung carcinogenesis model cell lines and smoking-related NSCLC cells. For the first time, our results demonstrated a link between the expression of miR-584-5p and smoking-induced methylation. Furthermore, we verified that miR-584-5p is controlled by methylation, as its expression was increased by demethylation. As shown in Figure 2D, the expression level of miR-584-5p was not increased by the 5-aza-dC treatment in 1799 cells, unlike what we observed in the 1198 and 1170I cells. This might be because the CpG sites of the miR-584-5p promoter were hardly methylated in 1799 cells exposed only to beeswax without CSC exposure [41]. Further, when we confirmed the effect of miR-584-5p on the invasion of the lung carcinogenesis model cell lines, an overexpression of miR-584-5p inhibited the invasion of 1170I cells but not 1198 cells (Figure 2F). We suspect that this is because 1198 and 1170I cells have different morphological characteristics. In addition, when we examined CpGs regulating the expression of miR-584-5p, we found that region 5 is a major site for regulation through methylation.

We also demonstrated that the overexpression of miR-584-5p could inhibit the migration and invasion of smoking-related NSCLC cells through its inhibitory effects on MMP-9. Additionally, this study is the first to show that miR-584-5p inhibits smoking-related NSCLC cell migration and invasion both in vitro and in vivo. To understand the mechanisms by which miR-584-5p inhibits migration and invasion in smoking-related NSCLC cells, we investigated a candidate target gene of miR-584-5p. According to previous reports, a single miRNA can regulate multiple target genes [42]. Likewise, we identified several candidate target genes for miR-584-5p, including Ras-related protein Rap-2a (RAP2A), ENAH, histone deacetylase 1 (HDAC1), and YKT6. However, among these candidate target genes, only YKT6 expression was significantly regulated by miR-584-5p at both the mRNA and protein levels in smoking-related NSCLC cells. Here, we showed that miR-584-5p could only inhibit migration and invasion but confirmed that miR-584-5p could also inhibit the proliferation of NSCLC cells (data not shown). However, YKT6 depletion could not significantly inhibit cell proliferation. Therefore, it is assumed that miR-584-5p may have other targets that inhibit the proliferation of NSCLC cells. Although this was not a prospective study, we found that a high expression level of YKT6 was associated with low survival rates in lung cancer patients with smoking experience by an analysis of the GEO database. Several previous studies have reported that YKT6 controls cell migration and invasion [43], and the level of this protein is significantly upregulated in p53-mutated tumors and in breast cancer cells resistant to docetaxel [44]. YKT6 is the target of miR-134 and miR-135b in NSCLC cells, and a low YKT6 expression has been reported to be associated with an improved survival of NSCLC patients [45]. YKT6, which is a SNARE protein recognition molecule, is involved in vesicular transport between secretory compartments [46] and is located in the membrane, cytosol, and perinuclear regions of cells. Due to its likely participation in various stages of intracellular vesicle trafficking, this SNARE protein might play essential roles in controlling the membrane dynamics during cell adhesion and migration [43]. Taken together, our results indicate that miR-584-5p, which functions as a tumor suppressor, was downregulated by methylation in smoking-related NSCLC cells. In addition, we demonstrated that the overexpression of miR-584-5p inhibited smoking-related NSCLC cell migration and invasion by targeting YKT6 both in vitro and in vivo. Therefore, these novel findings suggest that miR-584-5p is a potential molecular biomarker for smoking-related NSCLC.

## 5. Conclusions

In our study, we demonstrated that tumor suppressor miR-584-5p is an important factor in the developmental stage of smoking-related lung carcinogenesis. We found that miR-584-5p expression was downregulated by methylation. Moreover, overexpressed miR-584-5p suppresses the migration and invasion of smoking-related NSCLC cells by targeting YKT6 both in vitro and in vivo. In conclusion, tumor suppressor miR-584-5p can be used as molecular biomarker for smoking-related NSCLC.

## Figures and Tables

**Table 1:** Characteristics of the lung carcinogenesis model cell lines.

Cell Lines	Histological Stage ^a^	CSC Exposure	Tumorigenicity ^b^	Histology
1799	Immortalized	-	-	Adenocarcinoma
1198	Transformed	+	-	Adenocarcinoma
1170I	Tumorigenic	+	+	Adenocarcinoma

Abbreviations: CSC (Cigarette Smoke Condensate). ^a^ Lacroix et al. [18] and ^b^ Klein-Szanto et al. [19].

## Data Availability

Data is contained within the article or Supplementary Materials.

## Supplementary Materials

The following are available online at https://www.mdpi.com/2072-6694/13/5/1159/s1, Figure S1: Original images in Figure 4E for Western blots, Figure S2: Original images in Figure 7C for Western blots, Figure S3: Original images in Figure 7F for Western blots, Figure S4: Original images in Figure 8C for Western blots, Figure S5: Original images in Figure 8D for Western blots.
