# Supplementary material for: Tumor Suppressor miR-584-5p Inhibits Migration and Invasion in Smoking Related Non-Small Cell Lung Cancer Cells by Targeting YKT6
Source: Cancers (Basel). 2021 Mar 8;13(5):1159. doi: 10.3390/cancers13051159 (PMC7962648; doi:10.3390/cancers13051159)
Supplement: Supplementary file 1 [file cancers-13-01159-s001.pdf]

cancers

Supplementary Material: Tumor Suppressor miR-584-5p Inhibits Migration and Invasion in Smoking Related Non-Small Cell Lung Cancer Cells by Targeting YKT6

Saet Byeol Lee, Young Soo Park, Jae Sook Sung, Jong Won Lee, Boyeon Kim and Yeul Hong Kim

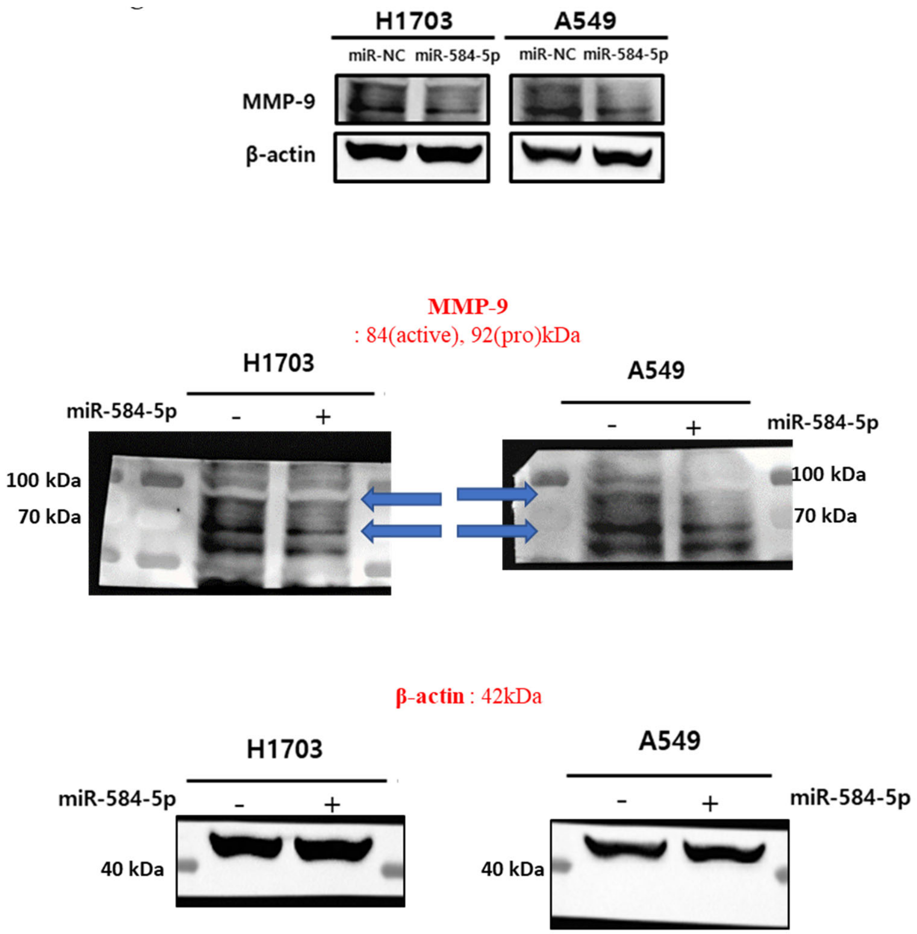

Figure S1. Original images in Figure 4E for Western blots.

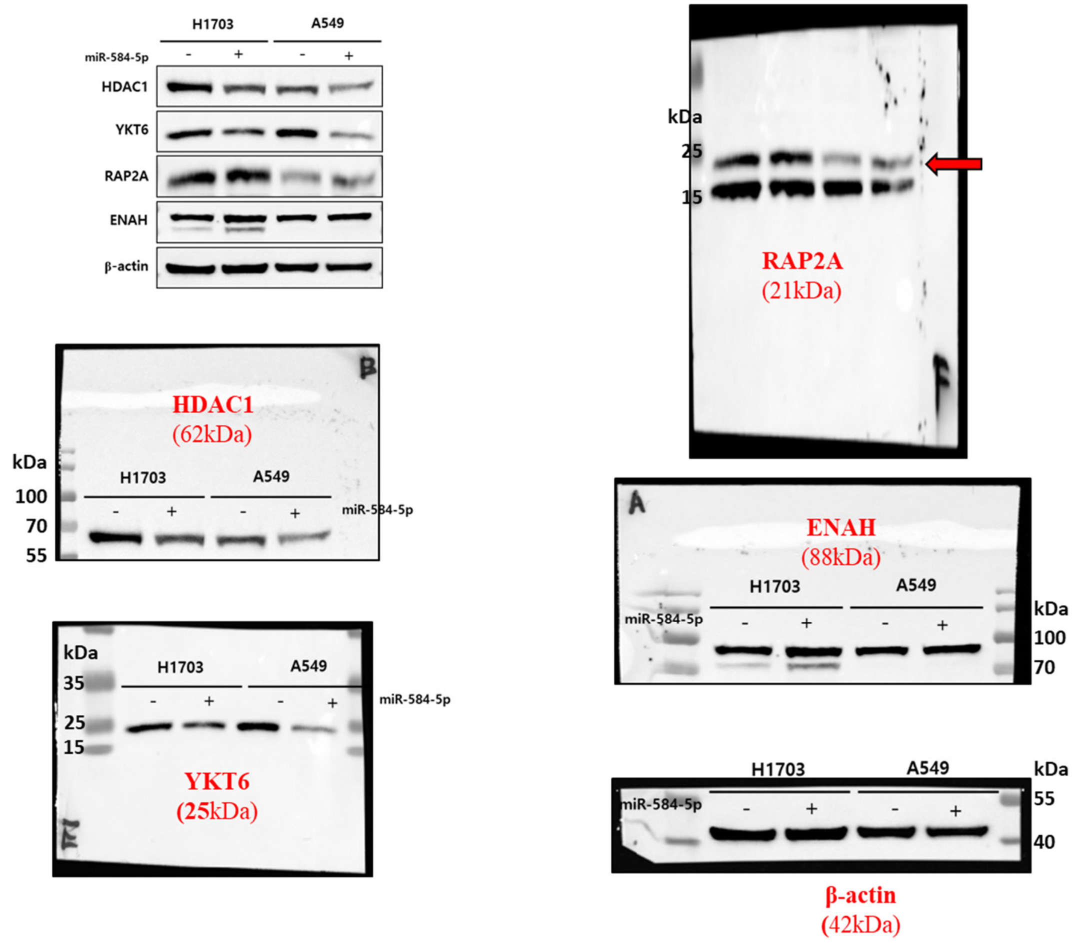

Figure S2. Original images in Figure 7C for Western blots.

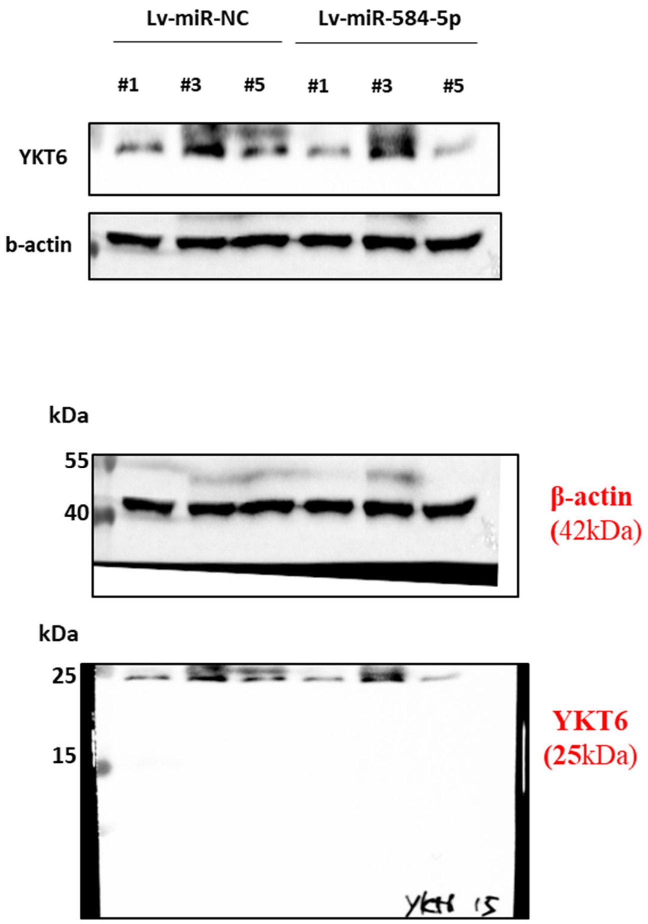

Figure S3. Original images in Figure 7F for Western blots.

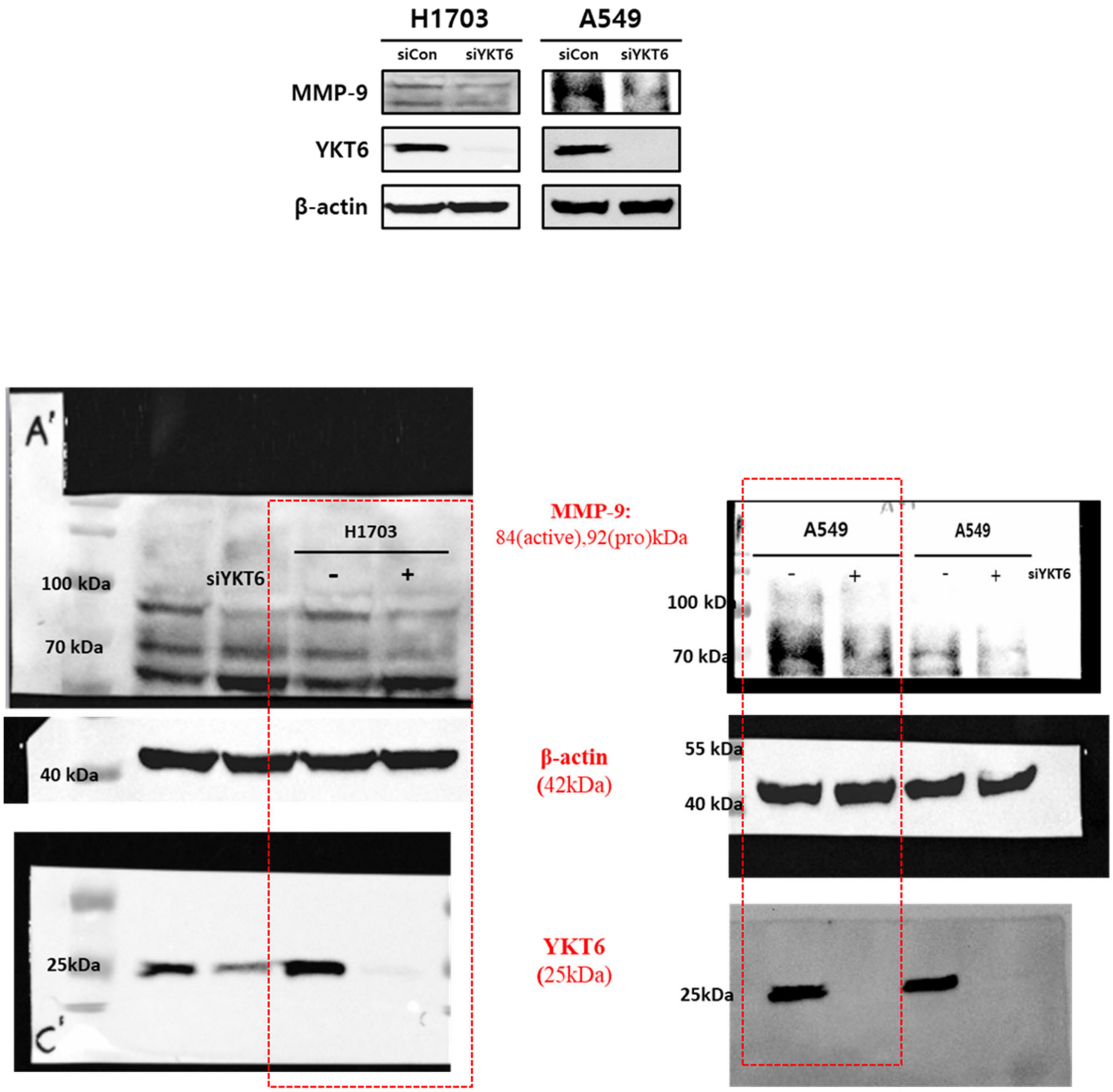

Figure S4. Original images in Figure 8C for Western blots.

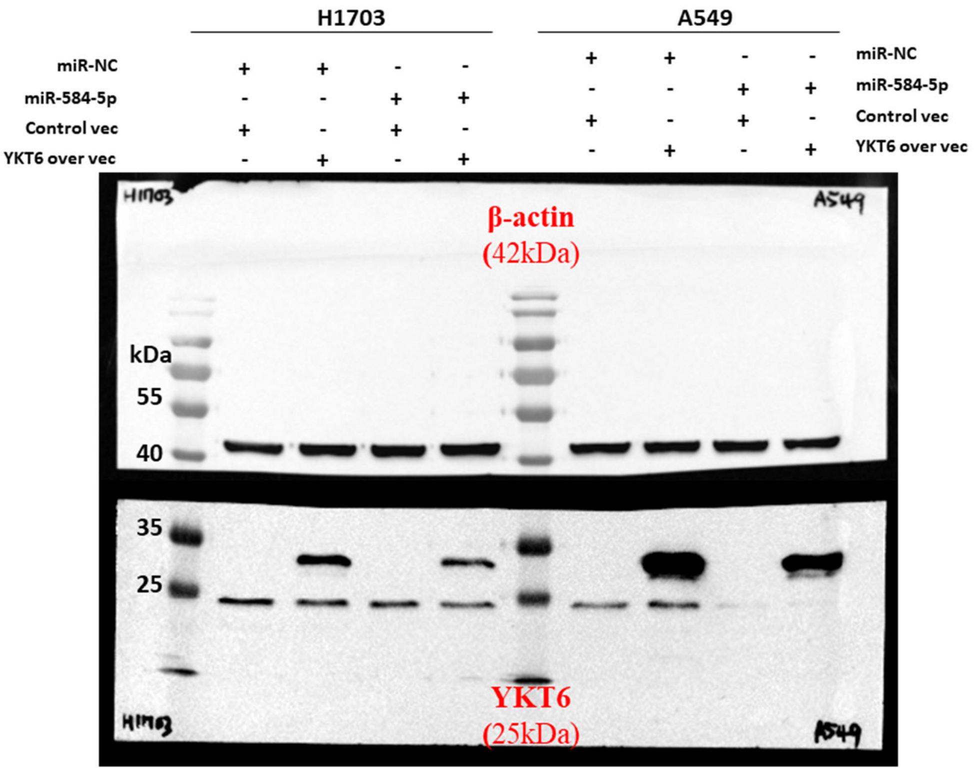

Figure S5. Original images in Figure 8D for Western blots.
